# Linguistic measures of personality in group discussions

**DOI:** 10.3389/fpsyg.2022.887616

**Published:** 2022-09-16

**Authors:** Lee A. Spitzley, Xinran Wang, Xunyu Chen, Judee K. Burgoon, Norah E. Dunbar, Saiying Ge

**Affiliations:** ^1^Department of Information Security and Digital Forensics, University at Albany, SUNY, Albany, NY, United States; ^2^Department of Management Information Systems, University of Arizona, Tucson, AZ, United States; ^3^Center for the Management of Information Systems, University of Arizona, Tucson, AZ, United States; ^4^Department of Communication, University of California, Santa Barbara, Santa Barbara, CA, United States

**Keywords:** language, personality, group communication, linguistic style, natural language processing

## Abstract

This investigation sought to find the relationships among multiple dimensions of personality and multiple features of language style. Unlike previous investigations, after controlling for such other moderators as culture and socio-demographics, the current investigation explored those dimensions of naturalistic spoken language that most closely align with communication. In groups of five to eight players, participants (*N* = 340) from eight international locales completed hour-long competitive games consisting of a series of ostensible missions. Composite measures of quantity, lexical diversity, sentiment, immediacy and negations were measured with an automated tool called SPLICE and with Linguistic Inquiry and Word Count. We also investigated style dynamics over the course of an interaction. We found predictors of extraversion, agreeableness, and neuroticism, but overall fewer significant associations than prior studies, suggesting greater heterogeneity in language style in contexts entailing interactivity, conversation rather than solitary message production, oral rather than written discourse, and groups rather than dyads. Extraverts were found to maintain greater linguistic style consistency over the course of an interaction. The discussion addresses the potential for Type I error when studying the relationship between language and personality.

## Introduction

The study of personality has a long history with most studies relying on self-report inventories of individual differences (see Montag and Elhai, 2019, for a historical review). One of the most dominant taxonomies of personality is the “Big Five of Personality” (McCrae and John, 1992) which is also known as the “Five Factor Model of Personality” (FFM; McCrae and Costa, 2004). The FFM is abbreviated with the acronym OCEAN which consists of these dimensions: Openness to Experience (experience seeking, education, reading widely, creativity, complexity), Conscientiousness (competence, order, dutifulness, self-discipline, dependability, and carefulness), Extraversion (warmth, gregariousness, assertiveness, enthusiasm, and positive affect), Agreeableness (trusting, straightforward, altruism, compliance, and sympathetic) and Neuroticism (anxiety, hostility, depression, self-consciousness, impulsiveness, and emotional instability; Gosling et al., 2003; Daly, 2011). Self-report measures require the design and administration of a survey, which is often disruptive and may have limited utility in predicting actual behavior (Boyd and Pennebaker, 2017). In many situations, especially those involving natural behavior, individuals will produce language that could indicate personality.

Studies of natural behavior and personality often examine the language that an individual produces. This technique has been used to infer personality from blog posts, written reflections, and day-to-day activities. Generally, these studies use language produced in solitary settings. Missing from the current literature is the role of personality in group conversation. Behavior in groups differ from monads and dyads (Levine and Moreland, 2012). Group members are not as obligated to speak as in monadic or dyadic situations (Pentland et al., 2021) and can easily engage in social loafing, which refers to a decline in individual effort in groups (Comer, 1995). With less obligation to speak, group members also have more time to prepare their speech should they decide to participate in the discussion. Moreover, when group members are motivated to speak and achieve their goals through persuasion, more presence of others during group conversations increases pressure, and individuals with certain personalities (e.g., introverts) may experience increased arousal and less smooth parallel processing if they speak in groups (Dewaele and Furnham, 1999). Within groups, participants may adopt roles, work to facilitate mutual understanding, and engage in impression management. As the technology to transcribe and process group discussion becomes more efficient and accessible, researchers will have the capability to estimate personality using language-based measures in group settings, rather than relying on self-reported data. We use this opportunity to examine relationships between personality and language production in a group setting to identify the boundary conditions of existing findings.

Daly (2011) identified several communication variables that pertain to personality that have been identified in past research and are likely very impactful in a group setting. Argumentativeness, the constructive sharing of ideas and being willing to debate them, is related to personality, as is verbal aggression, which is attacking and causing psychological pain during conflict. Compared to those who employ constructive argumentativeness, those who are verbally aggressive tend to be less open, more defensive, less flexible, and have poorer argumentation skills. Introverted individuals are also more prone to communication apprehension and conflict avoidance than extroverts. Inasmuch as our study involved a game that required conversation and debate, and shy people are less likely to talk in social settings (Garcia et al., 1991), the personality of the players should be evident through their language use and their use of nonverbal communication.

To understand personality through behavior, we conducted an experiment from a multi-cultural group interaction project in which small groups of participants (5–8 per group) participated in a multi-round, discussion-based decision-making game and completed multiple personality measures that allowed us to test whether their language patterns were influenced by personalities as compared to socio-demographics, task requirements and the like. It also allowed us to check the degree to which personality factors operated similarly or differently across the multiple cultures that were included in the sample.

Much of the prior work has used word-level measurements of written communication to measure personality. However, it is likely that personality manifests differently during interactive communication than it does when writing. There may also be useful, language-based measurements of personality at the sentence or utterance level of speech. We have access to natural language from many small group discussions that offer the opportunity to explore two questions:

1. What is the relationship between personality and language in the context of group discussion? Are existing techniques sufficient to measure personality in this setting?
2. Does linguistic style change over an interaction? Does personality moderate this?

This study contains several novel contributions to understanding the relationship between personality and language. Prior work on the relationship between personality and language has often focused on written texts that are especially amenable to study with dictionary-based analysis techniques. We seek to build on these findings in several ways. First, we are expanding analysis to a group context, which may alter participants’ proclivities to participate or not and amplify certain linguistic styles such as argumentativeness. Second, we examine spoken conversation, which may further capture participants’ typical discourse and offer a window into discourse dynamics. Third, we expand analysis to include additional measures of linguistic style that have not been covered in prior studies. These include composite measures (combinations of several features), such as lexical diversity, sentiment, and dominance, that may reflect more meaningful facets of style. Such language-based measures could lead to less expensive and less invasive measurement of personality (Boyd and Pennebaker, 2017).

## Literature review

### The relationship between language and personality

The relationship between language and personality has investigated questions of both language production and perceptions of personality through language (Gill, 2003). Studies on personality perception found that a variety of factors could influence the accuracy of a perceiver’s judgment of a target person’s personality, such as whether the perceiver is familiar with the target (Funder and Colvin, 1988) and whether the personality trait being judged is visible or hidden (Funder, 1995). Given the complexities of these factors, this study uses self-report instruments to measure personality and focuses on the first question of personality communicated through language production.

According to trait theory, personality refers to internal characteristics that determine behaviors (American Psychological Association Trait Theory, 2022). The behaviors in which personality is manifested cover many aspects of daily life, such as activities and locations as well as language use (Mehl et al., 2006). People’s language use is typically considered temporally stable and cross-situationally consistent in both written text (Schnurr et al., 1986; Pennebaker and King, 1999) and everyday speech (Mehl and Pennebaker, 2003), so language use satisfies the psychometric requirement for a personality variable (Ireland and Mehl, 2014). However, longer samples of discourse may be subject to contextually induced variability so that language exhibits some dynamic qualities as well.

Language use refers to content and style. Language content (e.g., percentage of words related to a topic) conveys an individual’s focus and meaning. According to the act frequency approach to personality, individuals with a trait engage in more acts that signal the trait (Buss and Craik, 1983), so the focus and meaning of their language reflects the behaviors, thoughts, and feelings congruent with their trait (Chen et al., 2020). Language style refers to the ways in which language is constructed irrespective of content, such as syntax choice and the use of function words (e.g., articles, pronouns) that form the grammatical structure. They are also useful markers of personality because function words convey information complementary to semantic meaning and indicative of traits (Ireland and Mehl, 2014). For example, the use of pronouns reflects shared knowledge between interactants and social cognition styles (e.g., attention to and interest in others) and hence may reveal personality traits like extraversion and agreeableness (Ireland and Mehl, 2014). Language styles are less constrained by the context and topic of communication. Whereas early studies on language production and personality focused on language content, researchers have called for more analysis of language styles (Ireland and Mehl, 2014). In a meta-analysis on predicting the Big Five personality dimensions with written language, Moreno et al. (2021) find the combined estimates of correlations are significant for all five traits, indicating that written language conveys personality. They also find that using both content and styles as predictors outperforms using either category.

Among the personality traits examined in relation to language production, extraversion is the most investigated trait; neuroticism is explored to a lesser extent; other traits are even less frequently investigated (Gill, 2003). One reason is that extraversion and neuroticism are the two less disputed traits, as they are common in the three-factor model of the Eysenck Personality Inventory (EPI; Katz and Dalby, 1981) and the five-factor model of personality (McCrae and Costa, 2004). Moreover, extraversion is a salient and highly visible trait (Funder, 1995). Overall, these studies demonstrate that individuals who score high or low on extraversion differ in their syntactic and semantic language behaviors. For example, extroverts tend to talk more (Gifford and Hine, 1994) and use more pronouns (including more second-person pronouns and more first-person plural pronouns), more verbs, and fewer prepositions in their speech (Dewaele and Furnham, 1999; Ireland and Mehl, 2014) than introverts. Extraversion is negatively associated with expressing tentativeness (e.g., perhaps), negations (e.g., never), exclusiveness (e.g., except, without), and inhibition (e.g., avoid) and positively associated with the use of words about positive emotion, social processes, leisure, and sex (Pennebaker and King, 1999; Ireland and Mehl, 2014; Chen et al., 2020). Dewaele and Furnham (1999) present a comprehensive review of linguistic features correlated with extraversion, and Chen et al. (2020) focus their meta-analysis on positive emotion and social process words as linguistic indicators of extraversion.

Neuroticism is found to correlate positively with immediacy in written texts, reflected by more use of first-person singular words (Ireland and Mehl, 2014) and fewer articles (Pennebaker and King, 1999). High neurotics also tend to convey more negative emotions (Pennebaker and King, 1999; Ireland and Mehl, 2014), more anxiety (Hirsh and Peterson, 2009), and negative affiliation to intimate relationships and groups (Gill, 2003) in written texts.

Linguistic behaviors associated with traits other than extraversion and neuroticism have gradually gained more academic attention. For example, Hirsh and Peterson (2009), Mairesse et al. (2007), and Lee et al. (2007) apply LIWC and its variants (i.e., a Korean version of LIWC in Lee et al. (2007)) to extract word categories that reflect topical content. They have identified several small to moderate correlations (absolute value typically smaller than 0.4) between LIWC language features and self-reported Big Five personality dimensions. Ireland and Mehl (2014) summarize this stream of literature and conclude that the linguistic markers of agreeableness include more positivity, more first-person singular pronouns, more words on social processes, home, family, and communication, and fewer words on death, money, and swearing. Indicators of openness include more articles and prepositions, fewer personal pronouns, and fewer words on family, home, and rest. Conscientiousness is negatively correlated with swearing and negative emotions.

Accompanying the study of linguistic behaviors is literature revealing the complexities of moderators. For example, public or private communication moderates the relationship between neuroticism and negative emotions because neurotic people are freer to express negativity in private than in public (Ireland and Mehl, 2014); moreover, the positive correlation between extraversion and positive emotions is stronger in public than in private, because extraversion is better revealed in public settings (Chen et al., 2020). Communication context, correspondent closeness, writing or speaking topic, gender, and facets of traits (e.g., the facets of intellect or artistic interests in openness) are also important moderators (Ireland and Mehl, 2014) which help explain some mixed findings in the literature.

## Hypotheses

The studies on language in written texts give us some prior background on what to expect in the relationship between language and personality. We develop hypotheses that include the findings of prior work, with some adaptation for group interactions. Our hypotheses primarily center around sentence-level linguistic features, especially those which were neither examined in prior work nor included in LIWC but might be informative of personality (Ireland and Mehl, 2014), thus providing additional predictive power beyond language content (Moreno et al., 2021).

### Lexical diversity

Lexical diversity measures the number of unique words in a passage of text. Dewaele and Furnham (2000) provide empirical evidence for a negative correlation between lexical diversity and extraversion in formal situations by analyzing speech from non-native French speakers taking a language exam. They attribute this relationship to extraverts’ lesser cognitive effort on lexical searching, so extraverts tend to use shorter high-frequency words. However, using private computer-mediated written text from English native speakers, Gill (2003) did not find such a negative correlation. It is likely that these mixed findings are because of various moderators, such as writing or speaking, spontaneity, private or public settings (Ireland and Mehl, 2014), and stress and cognitive demand. Our experiment involves spontaneous speech in public that requires cognitive effort for social deduction, so we hypothesize a negative association, which is consistent with Dewaele and Furnham (2000).

More conscientious individuals are likely to listen and attend to details (Witt and Ferris, 2003). In an interactive group setting, more conscientious participants may recall more details and use a more diverse lexicon as the discussion progresses. More anxiety leads to less lexical diversity in speech production (Höweler, 1972). Because neuroticism is positively correlated with anxiety (Muris et al., 2005), we hypothesize a negative relationship between neuroticism and lexical diversity. Openness to experience is highly correlated with fantasy, ideas, and esthetics (Compton, 1998). High openness individuals tend to be imaginative and broad-minded (McCrae and Costa Jr, 1983), so we hypothesize that these individuals display more lexical diversity to reflect their divergent thoughts.

*H1:* Lexical diversity is positively associated with (a) conscientiousness and (b) openness, and negatively associated with (c) extraversion and (d) neuroticism in group conversation.

### Expressivity

Expressivity reflects the use of adjectives and adverbs and is measured by the word count of adjectives and adverbs divided by the word count of nouns and verbs (Moffitt et al., 2012). Dewaele and Furnham (1999) found that non-native French speakers who are more extraverted use more adverbs and fewer adjectives in their spontaneous speech with a researcher. Although Gill (2003) argued extraverts’ language is less formal and hypothesized consistently with Dewaele and Furnham (1999) and Gill (2003) found the opposite (i.e., extraverts use fewer adverbs and more adjectives) with computer-mediated text written in private by English native speakers. These mixed findings may also be attributed to moderators, such as writing or speaking, spontaneity, and private or public communication (Ireland and Mehl, 2014). Because our experiment involves spontaneous speech in public, we expect extraverts to use more adverbs and fewer adjectives than introverts. Overall, we hypothesize extraverts’ language is more expressive. High openness individuals often show investigative interests (Connelly et al., 2014), so we expect them to articulate extra details in their speech by using more adverbs and adjectives.

*H2:* Expressivity is positively associated with (a) extraversion and (b) openness in group conversation.

### Complexity

Complexity refers to the use of big words and complex sentence structures. We operationalize lexical complexity by the number of words with six or more letters, the number of words with three or more syllables, and the average characters per word. Syntactic (sentence level) complexity is measured by common readability scores, including SMOG (Mc Laughlin, 1969), FOG (Gunning, 1969), and FRE (Crossley et al., 2011). We also use a complexity composite which accounts for both word and sentence level complexity (Burgoon et al., 2016). Conscientiousness is characterized by dutifulness and achievement striving (Yarkoni, 2011) and “interpersonally oriented behaviors that contribute to organizational goal accomplishment” (Van Scotter and Motowidlo, 1996). Because conscientious people listen more and attend to details (Witt and Ferris, 2003), they may need to reason through more information during their speech and thus display a more complex language style. Therefore, we hypothesize a positive relationship between conscientiousness and language complexity. One facet of openness is intellectual efficiency (Connelly et al., 2014), which refers to “individuals’ ability to process complex information.” Curiosity is another aspect of openness and shows “individuals’ interest in exploring and understanding novel information” (Connelly et al., 2014). Because our experiment settings require participants to collect and process information, we hypothesize that high openness individuals are more able to showcase their reasoning by reorganizing information in their speech, resulting in more complex sentence structures.

*H3:* Complexity is positively associated with (a) conscientiousness and (b) openness in group conversation.

### Immediacy

Immediacy is the extent to which an individual creates psychological closeness and takes ownership of a statement. Following Wiener and Mehrabian (1968) and Burgoon et al. (2016), we measure immediacy by the number of present-tense words, future-tense words, and first-person pronouns. Extraversion is positively correlated with the use of first-person plural pronouns, which reflects extraverts are sociable and talk with others frequently (Ireland and Mehl, 2014). Overall, there is no significant correlation between extraversion and the use of first-person singular pronouns, but this relationship may be moderated by the words following first-person singular pronouns (Ireland and Mehl, 2014). We hypothesize that extraverts use more immediate language than introverts. Agreeableness has been associated with other-centeredness and social interest (Compton, 1998), so more agreeable individuals may refer less to themselves. However, more immediate language could also reflect a closer emotional connection between an individual and others and a more agreeable personality. Empirically, both Pennebaker and King (1999) and Gill (2003) found a positive correlation between agreeableness and first-person pronouns, and the review by Ireland and Mehl (2014) was consistent with these two studies. Consequently, we hypothesize that more agreeable individuals refer more to themselves.

Highly neurotic individuals show more intense self-referential processing of negative stimuli (Cremers et al., 2010), which implies they tend to relate information from the external world to themselves. Empirically, both Pennebaker and King (1999) and Gill (2003) found a positive correlation between the use of first-person pronouns and neuroticism in private written text. In contrast, naturalistic spoken language (not necessarily in groups) does not show this relationship (Mehl et al., 2006), which may be attributed to the pressure from social norms (Ireland and Mehl, 2014). Because the group setting is an understudied moderator, we will test whether the positive correlation between immediacy and neuroticism extends to groups.

The relationship between openness and language immediacy could be twofold. On one hand, high openness individuals are more intellectually curious and self-reflective (Connelly et al., 2014), which could lead to a more immediate language style. On the other hand, high openness individuals tend to seek more diverse experiences from the external world (Connelly et al., 2014), resulting in less immediate language. Empirically, Pennebaker and King (1999) found people who score high on openness use less immediate language reflected by less use of first-person singular words and less use of present tense. Gill (2003) also found a negative correlation between openness and the use of present-tense verbs. Meanwhile, they identified a positive correlation between openness and first-person pronouns, and the inconsistency with Pennebaker and King (1999) could stem from differences between the corpora in use. We hypothesize a negative relationship between openness and language immediacy, because the presence of others in groups may prompt high openness individuals to seek more information from the external world.

*H4:* Immediacy is positively associated with (a) extraversion, (b) agreeableness, and (c) neuroticism, and negatively associated with (d) openness in group conversation.

### Dominance

Burgoon et al. (1998) propose that dominance is “a relational, behavioral, and interactional state that reflects the actual achievement of influence or control over another *via* communication actions.” To measure linguistic dominance, we use the total word count and the ratio of dominant turns-at-talk (e.g., those with “you must” or “I can”; Pentland et al., 2021). The opposite of dominance is submissiveness, which is measured by the amount of submissive turns-at-talk (e.g., seeking guidance and permission, negative self-evaluation; Moffitt et al., 2012). Extraversion is frequently associated with being sociable, talkative, and active (Barrick and Mount, 1991). Extraverts are found to write more words in computer-mediated communication (Gill, 2003). We hypothesize that extraversion is positively associated with verbal dominance because extraverts are expected to be more talkative and more actively involved in group conversation, which will lead to more verbal dominance. One facet of agreeableness is compliance (Costa Jr. and McCrae, 1995), which shows the tendency to defer to others and avoid conflicts. More compliance and less confrontation could lead to less verbal dominance (e.g., less “you must” and more permission-seeking), so we hypothesize that agreeableness is negatively associated with verbal dominance. Conscientiousness is characterized by achievement striving (Costa Jr. and McCrae, 1995) and interpersonal facilitation (Witt and Ferris, 2003). Conscientious individuals are expected to use more powerful and persuasive language to achieve their goals and engage more and speak more in group conversations. We hypothesize that conscientiousness is positively associated with verbal dominance. High openness individuals are more likely to be broad-minded and embrace different ideas (Costa Jr. and McCrae, 1995). More tolerance of different ideas could lead to less intent to persuade others, so we hypothesize that openness is negatively associated with verbal dominance.

*H5:* Verbal dominance is positively associated with (a) extraversion and (b) conscientiousness, and negatively associated with (c) agreeableness and (d) openness in group conversation.

### Uncertainty

Uncertainty refers to vagueness and fuzziness in one’s language (Burgoon et al., 2016). Following Burgoon et al. (2016), we measure uncertainty by hedging words, uncertainty quantifiers, uncertainty terms in the Loughran–McDonald (LM) dictionaries (Loughran and McDonald, 2011), and weak modals. Extraverts tend to be more assertive (Barrick and Mount, 1991)and use fewer tentative words (e.g., perhaps) and more certainty words (e.g., absolute) than introverts (Dewaele and Furnham, 2000; Yarkoni, 2011). Moreover, we expect high openness individuals to use hedge and uncertainty language to express their openness to ideas (Costa Jr. and McCrae, 1995).

*H6:* Uncertainty is positively associated with (a) openness, and negatively associated with (b) extraversion in group conversation.

### Sentiment

Sentiment refers to the affective or emotional state expressed in language. We measure sentiment by positive, negative, and neutral emotions extracted by existing text analysis tools, such as VADER (Hutto and Gilbert, 2014) and SentiWordNet (Baccianella et al., 2010). Other measures include word categories that are associated with positive or negative sentiment (e.g., Assent for positive sentiment, Sad and Death for negative sentiment). Extraverts are vigilant and sensitive to desirable and pleasant stimuli, so they tend to display positive emotion, which has been repeatedly confirmed by empirical studies (Chen et al., 2020). The review by Ireland and Mehl (2014) also finds a positive correlation between extraversion and positive emotion. Moreover, they identify linguistic positivity as a characteristic of agreeableness, which fits the definition of this personality trait (Gosling et al., 2003; Daly, 2011). We expect the language of high neurotics to be more negative. High neurotics convey more anxiety (Hirsh and Peterson, 2009) and negative affiliation to intimate relationships and groups (Gill, 2003) in written texts. Furthermore, the negativity may be moderated by groups. On one hand, high neurotics could take advantage of social loafing (Comer, 1995) and free riding and decrease their anxiety level, thus showing less negative sentiment. Second, the public nature of group conversations may further discourage neurotic individuals from freely expressing their negativity (Ireland and Mehl, 2014). On the other hand, more presence of others during group conversations increases pressure during speech and may cause high neurotic individuals to express more negativity. As our experiment settings motivate participants to actively speak and persuade their group members, individuals with high neuroticism may experience increased anxiety and become more negative in their speech if they decide to participate in the discussion. Ireland and Mehl (2014) claim in their review that conscientious people express less negative emotion and attribute this pattern to their self-regulation and conformity to social norms. Empirically, positive emotion is found to be associated with lower neuroticism, more extraversion, more agreeableness, and more conscientiousness (Pennebaker and King, 1999). Negative sentiment is found to be associated with higher neuroticism, lower agreeableness, and lower conscientiousness (Pennebaker and King, 1999). Mixed emotions and personality may also reflect personality (Barford and Smillie, 2016). For example, individuals with high openness have fewer extreme emotions, and their sentiment is perhaps positively related to neutrality from a measurement perspective. In summary, we hypothesize the following.

*H7:* Positive sentiment is positively associated with (a) extraversion, (b) agreeableness, and (c) conscientiousness, and negatively associated with (d) neuroticism in group conversation.

*H8:* Negative sentiment is positively associated with (a) neuroticism, and negatively associated with (b) agreeableness and (c) conscientiousness in group conversation.

*H9:* Neutral sentiment is positively associated with openness in group conversation.

*Articles.* Articles refer to both indefinite articles (i.e., “a” and “an”) and definite articles (i.e., “the”) and reflect linguistic formality. The use of articles is measured by the ratio of articles to total words in an utterance. Extraverts are eager to make verbal contributions in a dialogue, so they tend not to exert their efforts to organize their speech, and their language style is less formal (Gill, 2003). Some researchers have coined the term “lazy extraverts” to describe this tendency (Gill and Oberlander, 2003) which leads to less use of articles. Empirically, previous research identified a negative relationship between the use of articles and extraversion in written texts (Pennebaker and King, 1999). High neurotics’ susceptibility to anxiety could impair the functioning of their goal-directed attentional system (Eysenck et al., 2007) and manifest as less formality in speech. Previous research uncovered a negative relationship between the use of articles and neuroticism in written texts (Pennebaker and King, 1999; Yarkoni, 2011). High openness individuals’ intellectual curiosity may prompt them to discuss more about objects and events, resulting in more use of articles in their speech. Pennebaker and King (1999) and Yarkoni (2011) both find a positive relationship between openness and the use of articles in written texts.

*H10:* Use of articles is positively associated with (a) openness, and negatively associated with (b) extraversion and (c) neuroticism in group conversation.

### Negations

Negations refer to words like “no,” “not,” and “never” (Pennebaker and King, 1999). We operationalize negations by the ratio of negation words to total words in an utterance. Extraversion is found to negatively correlate with the use of negations (Pennebaker and King, 1999), which could be attributed to extraverts’ inclination to present positivity and appear sociable. Agreeableness is found to negatively correlate with the use of negations (Nowson, 2006), and this relationship may be ascribed to agreeable individuals’ tendency to trust others and less willingness to confront. Pennebaker and King (1999) and Yarkoni (2011) both find a negative relationship between conscientiousness and the use of negations, which may be attributed to conscientious people’s conformity to others’ requirements (Roccas et al., 2002). Neuroticism is found to positively correlate with the use of negations (Yarkoni, 2011), and the correlation reflects high neurotics’ general emotional negativity to environmental stimulation. Openness is found to negatively correlate with the use of negations (Yarkoni, 2011) because high openness individuals embrace diverse ideas.

*H11:* Use of negations is positively associated with (a) neuroticism, and negatively associated with (b) extraversion, (c) agreeableness, (d) conscientiousness, and (e) openness in group conversation.

Table 1 lists the measurement of the hypothesized linguistic variables. Table 2 shows the hypothesized relationships between personality and language variables. The plus sign indicates a hypothesized positive correlation; the minus sign indicates a hypothesized negative correlation.

**Table 1:** Measurement of language variables in the hypotheses.

Language variables	Measurement
Lexical diversity	The number of unique words in a passage of text
Expressivity	The word count of adjectives and adverbs divided by the word count of nouns and verbs
Complexity	Word level complexity: the number of words with 6 or more letters, the number of words with three or more syllables, and the average characters per word; Sentence level complexity: Simple Measure of Gobbledygook (SMOG; Mc Laughlin, 1969), FOG (Gunning, 1969), Flesch Reading Ease (FRE; Crossley et al., 2011), and a complexity composite which accounts for both word and sentence level complexity (Burgoon et al., 2016)
Immediacy	The number of present-tense words, future-tense words, and first-person pronouns
Dominance	The total word count and the ratio of dominant turns-at-talk (e.g., those with “you must” or “I can”; Pentland et al., 2021)
Uncertainty	The number of hedging words, uncertainty quantifiers, uncertainty terms in the Loughran-McDonald (LM) dictionaries (Loughran and McDonald, 2011), and weak modals
Positive Sentiment	Positive emotions extracted by VADER (Hutto and Gilbert, 2014) and SentiWordNet (Baccianella et al., 2010) and word categories that are associated with positive sentiment (e.g., Assent)
Negative Sentiment	Negative emotions extracted by VADER (Hutto and Gilbert, 2014) and SentiWordNet (Baccianella et al., 2010) and word categories that are associated with negative sentiment (e.g., sad, death)
Neutral Sentiment	Neutral emotions extracted by VADER (Hutto and Gilbert, 2014) and SentiWordNet (Baccianella et al., 2010)
Articles	The ratio of articles to total words in an utterance
Negations	The ratio of negation words to total words in an utterance

**Table 2:** Summary of hypotheses.

Language variables	Extraversion	Agreeableness	Conscientiousness	Neuroticism	Openness to experience	Hypotheses consistent with Ireland and Mehl (2014)
Lexical diversity	−		+	−	+	
Expressivity	+				+	
Complexity			+		+	
Immediacy	+	+		+	−	Extraversion, agreeableness, neuroticism (in private)
Dominance	+	−	+		−	
Uncertainty	−				+	Extraversion
Positive Sentiment	+	+	+	−		Extraversion, agreeableness
Negative Sentiment		−	−	+		Conscientiousness, neuroticism (in private)
Neutral Sentiment					+	
Articles	−			−	+	Openness to experience
Negations	−	−	−	+	−	

## Methods

### Participants

The research group decided to collect data from 100 participants at each locale before their global trips for data collection and ended up with 695 participants across eight sites. The difference between the planned and actual sample size was due to no-shows or technical issues. The data collection process resulted in 96 total games. We have obtained the transcripts for 47 games, which we use for this study.

Participants (*N* = 340; M_age_ = 22.84, SD_age_ = 3.91) were primarily college students, although some participants were recruited from the general public. Data collection took place at eight public universities in the Southwestern US (8 games; *n* = 55), Western US (6 games; *n* = 44), and Northeastern US (8 games; *n* = 56); international sites included Israel (5 games; *n* = 33); Singapore (7 games; *n* = 52), Hong Kong (5 games; *n* = 39), Fiji (5 games; *n* = 37), and Zambia (3 games; *n* = 24). The US sites were associated with the Principal Investigators. The international sites were among several who were invited to participate and who received institutional approval, government approval and who had a local host to coordinate data collection. Participants were recruited *via* email and advertisements on public message boards. The sample was 54% female, and was ethnically diverse (although this varied by location). Participants were required to be proficient English speakers and agreed to be video-recorded. We first obtained a rough transcription using IBM Watson automated speech-to-text. The automated transcripts contained errors and did not label speakers, so they were corrected and verified by human transcribers.

### Procedure

The data for this study were collected as part of a larger project investigating trust and deception in group negotiations. The data were collected between 2016 and 2018. Participants signed up for an experiment session using an online scheduling system. The sessions ranged from five to eight participants. After signing up, participants were sent an email with a unique identifier number and a link to a pre-survey, which included consent forms, cultural and psychological measures, and demographic questions. Upon arrival at the lab, participants were randomly assigned to one of eight computer stations that included a desk, a tablet with a built-in webcam, and a chair.

After all participants were seated, the experiment facilitator explained the rules of the game, and participants took part in an ice-breaker activity to get to know the other players. After this ice-breaker activity, players rated each other on several scales (see “Measures”). Participants took part in the game for an hour, during which they played between three and eight rounds. After the second, fourth, and sixth rounds, and at the end of the game, participants filled out several scales about their attitudes about the other players.

### Game play

**Figure 1:**
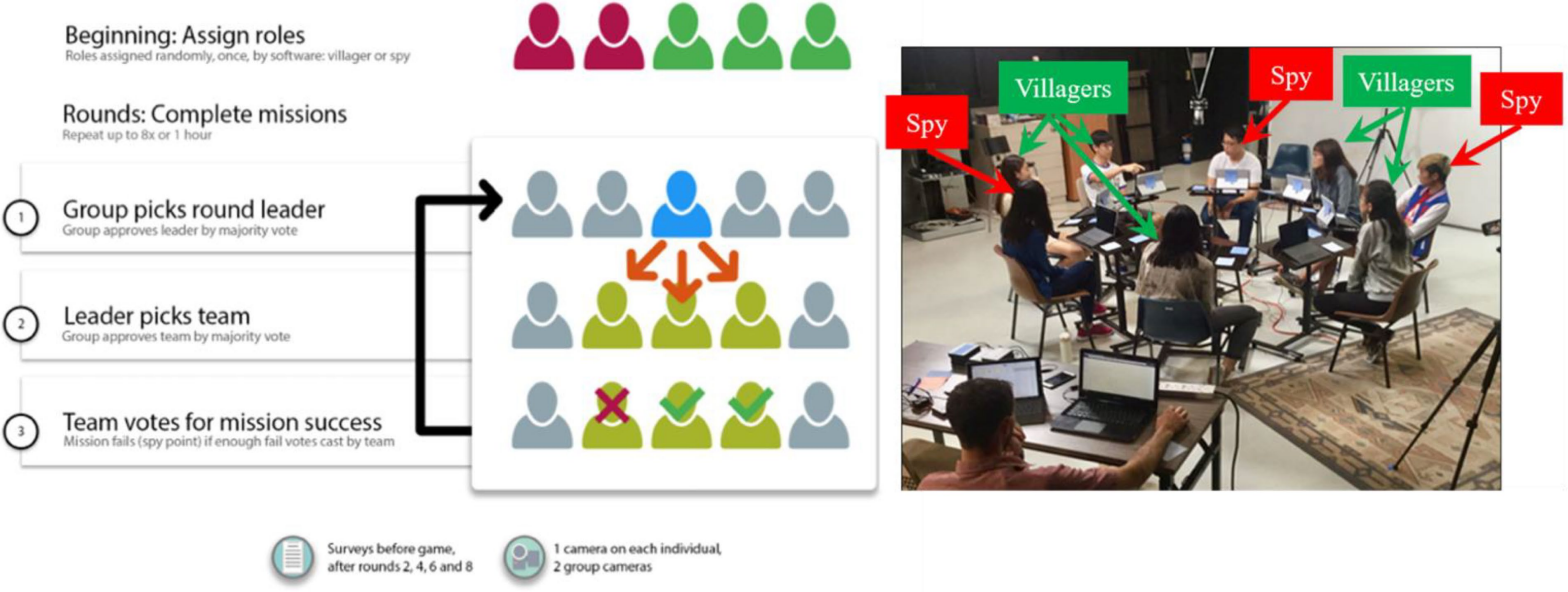
Game procedures.

Similar to Zhou et al. (2013), we employed a version of the *Mafia* game, but one that more closely resembles the board game *The Resistance*. We pilot-tested several versions of the game and adapted the rules to best address the research questions. Figure 1 shows the progression of the game and a picture of the physical layout. Players were randomly and secretly assigned to play deceivers (called “Spies”), or truth-tellers (called “Villagers”). In games of five or six players, two were assigned to be Spies, and in games of seven or eight players, three were assigned to be Spies. The Spies were aware of who the other Spies were, but the Villagers did not know anyone else’s role. Villagers had to depend on shared information to deduce the other players’ identities within the game.

Players completed a series of “missions” by forming teams of varying size. At the beginning of each round, players elected a leader, who then chose other players for these missions based on who they thought would help them win the game. All players voted to approve or reject the team leader, and then voted on the leader’s proposed team. Players voted secretly on their computer, and also voted publicly by raising their hands. They were allowed to vote differently on the computer than in public, and the facilitator would announce if there was a discrepancy in public and private votes, letting participants know that deception had occurred. Those who were chosen by the leader to go on the mission team secretly voted for the mission to succeed or fail. Villagers won rounds by figuring out who the spies were and excluding them from the mission teams. Spies won rounds by causing mission failures. The team that won the most rounds was the winner of the game. In addition to compensation for participating, players won monetary rewards by being voted as leader or winning the game.

Following each leader selection and team selection vote, the facilitator would reveal the voting result and encourage a discussion if the public vote did not match the private vote. These periods contained most of the language produced by the participants. After the facilitator announced the mission outcome, players provided their opinions on why they thought the mission resulted in the way it did. Facilitators were responsible for guiding the game process, clarifying confusion, and promoting additional discussion when groups were not sufficiently communicative.

### Measures

#### Game outcome

In Zhou et al.’s (2013) Mafia study, they operationalized deception detection success as the truth-tellers winning the game (i.e., if the truth-tellers win, they must have accurately detected deception). Similarly, in this study, game outcome was a dichotomous variable measuring whether Spies (*n* = 132) or Villagers (*n* = 208) won the game. Since there were more villagers than spies overall, they had a greater opportunity to win the game, but the outcome of the games overall was nearly a 50/50 split on spies and villagers winning.

#### Big 5 personality traits

As part of the pre-game survey, participants completed the Ten Item Personality Measure (TIPI) which is a short measure of the Big Five (or Five-Factor Model) dimensions, designed specifically for circumstances when very short measures are needed, personality is not the primary topic of interest, or researchers can tolerate the somewhat diminished psychometric properties associated with very brief measures (Gosling et al., 2003). The measure included extraversion (extroverted, enthusiastic; reserved, quiet–reverse-scored), agreeableness (sympathetic, warm; critical quarrelsome–reverse-scored), conscientiousness (dependable, self-disciplined; disorganized, careless–reverse-scored), neuroticism (calm, emotionally stable; anxious, easily upset–reverse-scored), and openness (open to new experiences, complex; conventional, uncreative–reverse-scored).

#### Additional variables

We collected several other variables during the pre-survey that we use as controls in this analysis. Specifically, all subjects were asked to complete an 18-item scale adapted from the Horizontal and Vertical Dimensions of Individualism and Collectivism (Singelis et al., 1995) and Park and Guan’s (2006) Positive and Negative Face scale to measure their cultural orientations. We also requested the subjects to report their first language. If they were not native English speakers, they were further asked when they started to learn English, and how many years they had been living in an English-speaking country. We also collected socio-demographic attributes, including gender, age, number of years of education completed, ethnicity and nationality.

## Results

### Software for linguistic analysis

We quantified language using LIWC2015 (Pennebaker et al., 2015) and SPLICE (Moffitt et al., 2012). LIWC uses dictionary-based measures of language and has been the primary method of inquiry into the relationship between language use and personality (e.g., Hirsh and Peterson, 2009; Yarkoni, 2011). SPLICE primarily uses part-of-speech tags to quantify language and offers additional measures of language style, such as syntactic complexity and lexical diversity. It has been used to study constructs like deception (Burgoon et al., 2016) and dominance (Pentland et al., 2021) and is a valuable tool for feature extraction to examine the relationship between personality and language styles in addition to those from LIWC. There are many similar measures between the tools. The counting of certain word types, like nouns, adverbs, and pronouns, should be relatively stable between the two. For both tools, count-based measures were divided by the total number of words.

Most studies of positive and negative emotion in relation to personality are based on LIWC categories. A limitation of this is that it does not consider context within a sentence, and it also does not consider the extremity of emotion, nor the use of neutral words. VADER (Hutto and Gilbert, 2014), a sentiment analyzer that considers sentence structure, e.g., saying something was “not good” may be scored as positive in a word-based analysis, though VADER would identify this as having negative valence. We also implement SentiWordNet *via* SPLICE. This uses weighted word counts to indicate the extremity of sentiment (positive, negative, or objective) for a word (Baccianella et al., 2010).

### Regression analysis

We used regressions to test our hypotheses. For each language measure, we ran linear mixed-effect regressions to find the marginal effect of the personality measures and to control for individual and game-level differences. The models were specified with each linguistic measure as a dependent variable, and personality measures as the independent variables. Sex, cultural measures, the participant’s role in the game (spy/villager) and whether they won or lost the game were controlled in the regression. Random effects were specified at the game level.

Table 3 shows the personality measures that have a significant marginal effect on a language measure. Extraversion was positively associated with the amount of verbal production (Word Count/Number of Words). Greater extraversion also predicted higher levels of LIWC positive emotion, Loughran-McDonald positive words, emotional tone, and affect words (e.g., happy, cried). Taken together, extraverts were emotionally expressive speakers. The ratio of hedging adjectives and characters per word were also positively associated with extraversion, suggesting that a higher level of uncertainty and complexity was expressed through the extraverts’ language. As a result, our prediction that extraversion is positively associated with dominance and positive sentiment are supported. However, no evidence was observed to support the other hypotheses on extraversion.

**Table 3:** Regression results for LIWC and SPLICE features.

	Extraversion	Agreeableness	Conscientiousness	Neuroticism	Openness
LIWC	Word count (+)^*^ Positive Emotions (+)^**^ Affect (+)^**^ Money (+)^*^ Emotional Tone (+)^**^ Friend (−)^*^ Discrepancy (+)^*^ Percept (+)^*^ See (+)^**^ Feel (+)^*^ Reward (+)^*^ Space (+)^*^ Home (+)^*^ Religion (+)^*^	Death (−)^*^ Affiliation (−)^*^ Female (−)^**^ She/He (−)^*^ We (−)^*^	Certain (+)^*^ [always, never] Discrepancy (+)^**^ [should, would] Cause (−)^*^ Function Words (+)^*^ Analytic (−)^*^ Body (+)^*^ Ingest (−)^*^	Money (−)^**^ Risk (+)^*^ Affiliation (+)^*^ [ally, friend, social] Drives (+)^*^ Cognitive Process (−)^*^ We(+)^**^	Ingest (+)^**^ [dish, eat, pizza] Assent (−)^*^ [agree, OK, yes] Friend (+)^*^
SPLICE	Number of Words (+)^*^ Hedge Adj (+)^*^ Verbs (+)^*^ Characters Per Word (+)^*^ LM Positive Count (+)^*^	Third Person Singular (−)^*^ FRE (+)^**^ FOG (−)^*^	Do Know (−)^*^ Nouns (−)^*^ Hedge All (+)^*^ SWN Positivity (+)^*^	Hedge Adv (−)^*^ First Person Plural (+)^**^ I Cannot Do It (−)^*^ SMOG (−)^*^	Agreement (−)^*^

The LIWC/SPLICE features in each cell are the features as the dependent variables that the corresponding personality factor was significantly associated with. Word Count (LIWC) and Number of Words (SPLICE) measured the total length of a speaker’s verbal messages. All other variables were averaged at utterance level. The sign in the round bracket indicates the direction of the personality factor’s effect. ^*^p < 0.05; ^**^p < 0.01. Content in the square bracket are the example words cited from the LIWC documentation. Features in bold measure the same or similar aspect of language but are named differently in their respective system.

Participants high in agreeableness had a lower proportion of third-person singular pronouns (she/he) and a smaller percentage of first-person plural pronouns, death, and affiliation words in their speech. Meanwhile, the readability score, FRE, was higher for the agreeable speakers, while FOG was smaller for them. As first personal plural commonly signals verbal immediacy, our prediction on the relationship between Agreeableness and Immediacy was not supported. However, if we assume that death words (e.g., kill, coffin) generally evoke negative feelings, our hypothesis that agreeableness leads to less negative sentiment was supported. The other agreeableness-related hypotheses were unsupported.

The LIWC models show that conscientious subjects used more certain and discrepancy words. In the experiment, these words were often used when making suggestions. However, the SPLICE models reached mixed results as they report that the ratio of hedging words, which signals verbal uncertainty, is positively associated with conscientiousness. The prediction that conscientiousness is positively related to the expression of positive sentiment is somewhat supported by the SentiWordNet positivity measure, but extraversion was overall a stronger predictor of positive sentiment. No other hypotheses on conscientiousness were supported.

For neuroticism, both the LIWC and SPLICE models indicate that subjects scored higher on this dimension used a higher percentage of first-person plural in their verbal messages, suggesting a positive connection between neuroticism and verbal immediacy. However, no intuitive link can be developed between the other significant LIWC/SPLICE features and our developed hypotheses.

Finally, the most significant finding on openness is its negative association with the percentage of assent words (e.g., agree, OK, yes), which we may consider as an indicator of positive sentiment. However, none of our hypotheses on openness were supported.

### Canonical correlation analysis

We also ran canonical correlations due to the possibility of overlap within the many variables under examination. When groups of variables are thought to be theoretically interrelated, as with Personality variables forming the construct Personality or linguistic variables forming interdependent dimensions of language, a good statistical method to use is canonical correlation, which shows the relationship between sets of variables. In this case, it is between the personality measures on the one hand and the verbal signals on the other hand. The purpose is to find linear combinations of X (the personality measures) and Y (the verbal signals) that have maximum correlation with each other. The result is a multiple correlation. The method is only appropriate with large sample sizes, which is the case here.

We first analyzed the SPLICE-based measures. We removed 11 measures with zero or near-zero values. To accomplish data reduction, reduce multicollinearity (e.g., multiple measures of “FOG”), and achieve greater clarity, we conducted Pearson product–moment bivariate correlations, then we conducted exploratory factor analysis (EFA) with Varimax rotation to combine measures into understandable dimensions of language. An eight-factor solution accounting for 52% of the variance produced these factors:

- *Pleasantness/Imagery* (Activation, Pleasantness, Imagery, SWN Objectivity and Number of Adverbs—Number of Nouns and Singular Mass Nouns)
- *Complexity* (Complexity composite, SMOG index, FOG index, Pausality, LWRF, Conjunctions—Lexical Diversity)
- *Big Words* (Syllables/Word, Number of Words with 6 or more Letters, Number of words with three or more syllables, Total Dominance, Accrued Dominance, Characters/Word, Number of Adjectives)
- *Uncertainty/Hedging* (Weak Modals, LM Uncertainty, Number of Speculative Words, Hedging Adverbs, Uncertainty Hedges)
- *Nonfluencies* (Number of UmUh, Number of Um, Hedging Verbs, First Person Singular, Total Hedges, Total Disfluencies)
- *VADER/Agreement* (Positive VADER Average, Number of Interjections, Number of Agreements, VADER Compound Average)
- *Immediacy* (Number of Verbs, Present Tense – Vader Negative Average and Repeat Phrases)
- *Submissiveness* (Accrued Submissiveness, “Do not Know” Expressions, Total Submissiveness)

These eight dimensions were then analyzed with canonical correlation, with the five personality dimensions forming Set 1 and the eight linguistic dimensions forming Set 2. As can be seen in Table 4, none of the vectors formed from the combination of features is significant.

**Table 4:** Canonical correlations among five personality measures and eight dimensions formed by linguistic features.

	Correlation	Eigen value	Wilks statistic	*F*	Numerator *df*	Denominator *df*	Sig.
1	0.223	0.053	0.884	1.018	40	1423.796	0.440
2	0.189	0.037	0.931	0.848	28	1180.437	0.694
3	0.136	0.019	0.965	0.650	18	928.209	0.861
4	0.120	0.015	0.983	0.556	10	658.000	0.850
5	0.048	0.002	0.998	0.190	4	330	0.943

Because the TIPI has 4 degrees of freedom, conceivably it could be decomposed into four different linear vectors that could be correlated with various linear combinations of the verbal signals. However, our interest in the relationships in the smallest subsets of combined personality-verbal signal sets is defeated by the lack of statistical significance for the four vectors. An examination of the proportion of variance accounted for (Table 5) reveals that Set 2 Predicted by Set 1, and Set 1 predicted by Set 2 shows very little common variance.

**Table 5:** Proportion of variance accounted for among set of personality dimensions and set of linguistic features.

Canonical variable	Set 1 by self	Set 1 by Set 2	Set 2 by self	Set 2 by Set 1
1	0.191	0.010	0.145	0.007
2	0.207	0.007	0.079	0.003
3	0.240	0.004	0.136	0.003
4	0.191	0.003	0.144	0.002
5	0.172	0.000	0.103	0.000

The LIWC variables were likewise submitted to EFA with Varimax rotation. However, a nine-factor solution (after removal of variables with low loadings) produced several uninterpretable factors. Moreover, some variables such as Causation, We, and He/She that represent frequently relevant variables such as personal pronouns were omitted. To uncover specific individual variables that correlate with the individual personality dimensions, all LIWC variables were analyzed with simple Pearson bivariate correlations with bootstrapping (*N* = 1,000).

Overall, very few significant correlations emerged. The greatest number of significant correlations emerged for Extraversion, but none exceeded *r =* 0.22. The other personality variable to show some relationship to language was Conscientiousness (see Table 6) but, like Extraversion, showed very weak relationships, with no correlation larger than 0.21. These correlations were small enough that they might be due to Type II error and/or indicate very modest relationships between language variables and personality.

**Table 6:** Language variables with significant correlations with personality dimensions.

Personality measure	Linguistic variable	Correlations significant *p* < 0.01	Correlations significant *p* < 0.05	Source
Extraversion	Word count	0.15		LIWC
	Affect	0.21		LIWC
	Positive emotion	0.20		LIWC
	Anger		0.12	LIWC
	Perceptual terms		0.13	LIWC
	See		0.12	LIWC
	Feel		0.12	LIWC
	Swear words		0.12	LIWC
	Netspeak		−0.12	LIWC
	Fillers		−0.11	LIWC
	Tone	0.18		LIWC
Agreeableness	Complexity composite, SMOG index, FOG Index, Pausality, LWRF, conjunctions—Lexical diversity		−0.12	SPLICE
Conscientiousness	Function words		0.13	LIWC
	Pronouns		0.13	LIWC
	Personal pronouns		0.12	LIWC
	Auxiliary verbs		0.13	LIWC
	Verbs		0.17	LIWC
	Numbers		−0.11	LIWC
	Discrepancy		0.14	LIWC
	Exclamation		0.13	LIWC
	Parenthetical remark	0.19		LIWC
Openness	Pleasantness/imagery (activation, SWN objectivity, number of adverbs–number of nouns)		0.13	SPLICE

What are we to make of these findings? One possibility is that there is a null relationship between language and personality, although there is enough counter evidence to challenge this conclusion. Another possibility is that language is highly dynamic, especially in a group setting, changing enough over time that it is not possible to draw conclusions about any one individual at any single point in time. In other words, an individual’s characteristic language pattern may be unique for that person but dynamic enough that it cannot be captured by a single snapshot. A third possibility is that the measures included here are simply not sensitive enough to capture personality beyond Extraversion.

It also should be evident from the variability across the SPLICE and LIWC measures that a single coding system is inadequate to represent an individual’s linguistic discourse. LIWC has greater breadth, but its simple counts of features are inadequate to capture the nuances of discourse and it has significant redundancy across its categories at the risk of Type I error. It also conflates topical content categories with syntactic ones, and SPLICE aims to reduce Type I error by combining indices into theory-driven categories but consequently may risk omitting other relevant features.

### Summary of findings

Based on the analysis above, we found that many of our hypothesized relationships were not supported.

*Lexical diversity:* H1(a), H1(b), H1(c), and H1(d) are not supported.

*Expressivity:* H2(a) and H2(b) are not supported.

*Complexity:* H3(a) and H3(b) are not supported. We identify a positive relationship between Extraversion and complexity (measured by characters per word) using regression analysis. The relationship between agreeableness and complexity shows mixed results. On one hand, correlation analysis shows complexity is negatively correlated with agreeableness. On the other hand, regression analysis shows two measures of complexity (FRE and FOG) are significantly associated with agreeableness with opposite directions. The regression analysis shows that complexity (measured by SMOG) is negatively correlated with emotional stability.

*Immediacy:* H4(a) is not supported. We find a negative relationship between the use of first-person plural pronouns and Agreeableness, so H4(b) is not supported. We find a positive relationship between the use of first-person plural pronouns and Emotional Stability with regression analysis, so H4(c) is supported. H4(d) is not supported.

*Dominance:* Both correlation and regression analysis show Extraverts speak more, which supports H5(a). H5(b), H5(c), and H5(d) are not supported.

*Uncertainty:* H6(a) is not supported. From the regression analysis using SPLICE variables, we identify a positive correlation between uncertainty (measured by the percentage of hedging adjectives) and Extraversion, which is opposite to H6(b). Regression results show mixed relationships between uncertainty and conscientiousness. Regression also shows the use of hedge adverbs is negatively associated with Emotional Stability.

*Sentiment:* Positive emotion words in LIWC were associated with greater Extraversion, and SentiWordNet Positivity was associated with greater Conscientiousness, which supports H7(a) and H7(c). However, this was not the case with VADER sentiment. This could be the result of the nuances captured by the VADER algorithm. Natural language tends to contain more positive words, but sentence structure and negations are often used to express negative or neutral emotions even with positively valenced words. H7(b) and H7(d) are not supported. We also find Openness is negatively associated with positive sentiment (measured by the Assent word category and the percentage of agreement words).

H8(a) is not supported. Regression analysis shows a negative relationship between agreeableness and negative sentiment (measure by the Death word category), so H8(b) is supported. H8(c) is not supported.

H9 is not supported.

*Articles and negations:* H10 and H11 are not supported.

### *Post hoc* analysis: Linguistic style dynamics

Given the relatively weak findings in our primary analysis, one possible explanation is that group members adapt their speech to conform to group norms. Prior work on personality and language has reported that linguistic measures of personality are relatively stable over multiple observations (Yarkoni, 2011). However, findings from dyadic communication indicate that language style likely changes over an interaction, as individuals may adjust their style to more closely match their conversation partner (Niederhoffer and Pennebaker, 2002). This seems to occur in groups, too. Review of group communication processes shows that language convergence is an important part of group performance (Van Swol and Kane, 2019). Thus, participants may have adjusted their language to the group and made it more difficult to detect distinct personalities. There may also be personality traits that moderate the extent to which individuals mimic the rest of the group rather than adhere to their own stable style.

Linguistic style matching usually measures style matching between individuals, but it can also measure linguistic style consistency by comparing within participants over the course of the game. We measured language style consistency by comparing participants to themselves in the previous round, beginning with the self-introductions. That is, a participant’s language in round 1 is compared to their language from the introduction, in round 2 their language is compared to round 1, etc. We used three different subsets of linguistic features to compute similarity. One uses the hypothesized measures in our study, another uses measures from Ireland et al., (2011), and one uses function words with pronouns excluded. Each measure yielded nearly identical results. Language similarity was measured using cosine similarity because the Ireland similarity measure is no longer guaranteed to produce values between 0 and 1 if we wish to use ratio-based measures and VADER sentiment (which ranges from [−1, 1]). This measure represents language measures as a multidimensional vector, and similarity is computed as the cosine of the angle between the two vectors. This method is commonly used to compare texts and is not subject to differences in text length.

After getting style consistency for each participant, we used hierarchical mixed-effect regressions to evaluate the ability of each personality dimension to predict language style consistency in the game. These regressions were specified like the regressions used in the previous section with the same control variables. The dependent variable is a participant’s language style consistency as compared to their own language in the previous round of the game. The resulting models are reported in Table 7.

**Table 7:** Effect of personality on style consistency.

	Dependent variable:	
	Cosine similarity	
	(1)	(2)
		
Extraversion	0.023^***^	0.027^***^
	(0.006)	(0.007)
Agreeableness	−0.015^*^	−0.009
	(0.008)	(0.009)
Conscientiousness	0.008	0.009
	(0.008)	(0.008)
Emotional Stability	−0.002	−0.004
	(0.007)	(0.008)
Openness	−0.008	−0.006
	(0.009)	(0.009)
Game Round	0.013^**^	0.012^**^
	(0.005)	(0.005)
Sex = Male	0.094^***^	0.095^***^
	(0.019)	(0.019)
Non-native English Speaker	−0.052^**^	−0.049^**^
	(0.021)	(0.021)
Villager		0.020
		(0.018)
Winner		0.024
		(0.018)
Horizontal Collectivism		0.003
		(0.013)
Horizontal Individualism		−0.005
		(0.011)
Vertical Collectivism		−0.013^*^
		(0.007)
Vertical Individualism		0.002
		(0.006)
Negative Face		−0.005
		(0.012)
Positive Face		−0.010
		(0.011)
Constant	0.699^***^	0.759^***^
	(0.075)	(0.106)

Coefficients are provided for each measure, with standard deviations in parentheses.

* *p* < 0.1;

** *p* < 0.05; and

*** *p* < 0.01.

There were 995 player/round observations. In the first regression, which did not contain controls for culture, Extraversion had a significant positive effect on style consistency over the course of the game, and agreeableness had a small, negative effect that disappeared when adding controls. Males had significantly greater language style consistency over the course of the game. For participants where English was not their native language, style consistency was significantly lower. The model also contained controls for time (“round”), and this had a positive association with style consistency as the game progressed.

To check the validity of this measure, we also ran a model where the dependent variable was a participant’s similarity to a randomly selected player in the same game during the same round. In this model, there were no variables that were significant at *p* < 0.05. Participants did have higher LSM scores as games progressed (*r* = 0.112, *p* < 0.001), but self-reported personality measures did not have any explanatory power.

## Discussion

In this study, we evaluated new and existing linguistic measures that may be associated with personality. There were modest relationships between language and the personality measures in our study. Many of our hypotheses, derived from prior work on language, cognition, and small groups, were not supported, though we did confirm some findings on word count and pronoun usage. This was surprising, given the rich history of findings in personality and language research and our relatively large sample. There are several possible explanations for this. First, group conversation is a complex and dynamic setting, which may lead to greater variance in personality expression versus written texts often used in prior research. Participants in our study were tasked with the goal of winning a competitive game, and likely adjusted their speaking patterns to achieve that goal. In addition, the game itself probably limited the types of words that were used in the discussion. The “death” (kill, coffin) words were unlikely to come up in this context, but other words like vote, ballot, spy, villager, and mafia (the name of the game) likely came up an unusual amount. Indeed, we find that linguistic style matching between participants increases during the interaction as they adapted to one another and zeroed in on conversational styles and topics—possibly diminishing the effect of personality expression through language.

Another possibility is that personality is expressed through content rather than linguistic style. Many of the LIWC categories that showed significant relationships were topical (i.e., money, ingest, affiliation, etc.). Evidence from financial earnings calls also shows the ability to predict personality from linguistic content (Harrison et al., 2019). We do not want to understate the importance of syntax. While our results around emotion were similar to prior findings, we did not find any significant relationship between VADER sentiment and personality. This may be due to the complex expression of emotion that often includes the context of the phrase, negations, and other modifiers. When looking at style through language style matching, we find that individuals adapt their style to that of the group. Extraversion had a positive association with style consistency (i.e., language that is like the individual’s language in prior game rounds). This may be driven by Extraverts’ tendency to use more words, which could lead to other members of the group mimicking those who speak the most.

Third, our findings are likely to be somewhat conservative. We tested our hypotheses using a regression analysis with all five personality measurements and numerous control variables. This differs from much of the prior literature, which is based on correlational analysis (e.g., Pennebaker and King, 1999; Hirsh and Peterson, 2009; Yarkoni, 2011). As a result, the measures that we have identified are less likely to be the result of Type I error than what was previously reported. There is still some risk when using many regression models to test hypotheses. In a supplementary document we compare our findings as reported here with multiple value of p corrections. After corrections, fewer measures were significant. However, these corrections increase the likelihood of Type II error. Given the historical reliance on correlational analysis of the relationship between language and personality, we are already reducing Type I error with regression and canonical correlation. This is demonstrated by our own correlational analysis compared to the regressions.

We have included a supplement with a correlation-based analysis that shows a substantial amount of statistically significant correlations disappeared when tested using regressions. To further contextualize our findings, we conducted a power analysis. The data collection was designed to detect medium effects. With this sample (340 participants) one can detect an effect of *r* = 0.151 with 0.8 power. The correlations from Kern et al. (2014) suggests effects between |0.023| < *r* < |0.124|. We also calculated the required N to have 0.8 power for the same range of effect sizes. These would require 507 < N < 14,835. Also, our sample included participants from diverse cultural backgrounds, and many were non-native English speakers. Their fluency might have affected their use of modifiers, the complexity of their sentences, and their correct usage of verb tenses.

## Conclusion

This study has provided several important contributions to the study of personality and language use. This is among the first to use language generated during a group interaction. We also introduce interaction-based measures (LSM), new measures and controls rather than simple correlations to improve the likelihood we are seeing real connections between language and personality traits. It may be disappointing to see that to a large extent, Extraversion, Agreeableness, and Neuroticism were the only personality traits related to language features, but when studying deception, as we were in this context, what *does not* work is just as important as what *does* work. Extraverts were more positive, more uncertain, used more complex words, and spoke more than other personality types. Agreeable personality types were less likely to use first-person plural pronouns and express negative sentiment. Neurotic individuals tended to use more first-person plural pronouns and less complex and uncertain language. With few other exceptions, these three personality types (Extraversion, Agreeableness, and Neuroticism) were more indicative of linguistic styles than any others. Our new measures and improved feature analysis as well as longitudinal measurement should be useful to other researchers to explore in other contexts.

Future research on personality measurement from group discussions should also consider several study design factors. This technique could be used in another setting where more data is available, such as an online forum or public discussions (like financial earnings calls or debates). It could also be used in a more standardized setting like video conferencing to reduce complexity compared to face-to-face interaction. Syntactic features have received little attention but could be useful in measuring personality from an individual’s text, like written statements or blog posts. Future work on personality could also use statistical tests that consider the marginal contribution of each personality dimension on language use.

There are several opportunities for future studies on language, personality, and group interactions. One line of inquiry could consider the role of personality on group-level outcomes, like productivity or cohesiveness. The context of our study was an adversarial situation—a cooperative group activity may result in more genuine behavior from all individuals in the group.

## Data availability statement

The datasets presented in this article are not readily available because privacy restrictions exist and data is not available until the end of grant. Requests to access the datasets should be directed to JB (judee@arizona.edu).

## Ethics statement

The studies involving human participants were reviewed and approved by the Institutional Review Board (IRB) at UC Santa Barbara. The patients/participants provided their written informed consent to participate in this study.

## Author contributions

All authors listed have made a substantial, direct, and intellectual contribution to the work and approved it for publication.

## Funding

This work was supported in part by a grant from Army Research Office grant W911NF-16-1-0342 (PI: JB).

## Conflict of interest

The authors declare that the research was conducted in the absence of any commercial or financial relationships that could be construed as a potential conflict of interest.

## Publisher’s note

All claims expressed in this article are solely those of the authors and do not necessarily represent those of their affiliated organizations, or those of the publisher, the editors and the reviewers. Any product that may be evaluated in this article, or claim that may be made by its manufacturer, is not guaranteed or endorsed by the publisher.

## References

[ref1] American Psychological Association Trait Theory. (2022). APA dictionary of psychology. Available at: https://dictionary.apa.org/trait-theory (Accessed May 10, 2022).

[ref2] Baccianella S. Esuli A. Sebastiani F. (2010). “Sentiwordnet 3.0: An enhanced lexical resource for sentiment analysis and opinion mining,” in *Proceedings of the Seventh International Conference on Language Resources and Evaluation (LREC’10)*.

[ref605] Barford K. A. Smillie L. D. (2016). Openness and other Big Five traits in relation to dispositional mixed emotions. Pers. Individ. Differ. 102, 118–122.

[ref3] Barrick M. R. Mount M. K. (1991). The big five personality dimensions and job performance: a meta-analysis. Pers. Psychol. 44, 1–26. doi: 10.1111/j.1744-6570.1991.tb00688.x

[ref4] Boyd R. L. Pennebaker J. W. (2017). Language-based personality: a new approach to personality in a digital world. Curr. Opin. Behav. Sci. 18, 63–68. doi: 10.1016/j.cobeha.2017.07.017

[ref5] Burgoon J. K. Johnson M. L. Koch P. T. (1998). The nature and measurement of interpersonal dominance. Commun. Monogr. 65, 308–335. doi: 10.1080/03637759809376456

[ref6] Burgoon J. K. Mayew W. J. Giboney J. S. Elkins A. C. Moffitt K. Dorn B.. (2016). Which spoken language markers identify deception in high-stakes settings? Evidence from earnings conference calls. J. Lang. Soc. Psychol. 35, 123–157. doi: 10.1177/0261927X15586792

[ref7] Buss D. M. Craik K. H. (1983). The act frequency approach to personality. Psychol. Rev. 90, 105–126. doi: 10.1037/0033-295X.90.2.105

[ref8] Chen J. Qiu L. Ho M.-H. R. (2020). A meta-analysis of linguistic markers of extraversion: positive emotion and social process words. J. Res. Pers. 89:104035. doi: 10.1016/j.jrp.2020.104035

[ref9] Comer D. R. (1995). A model of social loafing in real work groups. Hum. Relat. 48, 647–667. doi: 10.1177/001872679504800603

[ref10] Compton W. C. (1998). Measures of mental health and a five factor theory of personality. Psychol. Rep. 83, 371–381. doi: 10.2466/pr0.1998.83.1.371, PMID: 9775694

[ref11] Connelly B. S. Ones D. S. Davies S. E. Birkland A. (2014). Opening up openness: a theoretical sort following critical incidents methodology and a meta-analytic investigation of the trait family measures. J. Pers. Assess. 96, 17–28. doi: 10.1080/00223891.2013.809355, PMID: 23819531

[ref604] Costa Jr P. T. McCrae R. R. (1995). Domains and facets: Hierarchical personality assessment using the Revised NEO Personality Inventory. J. Pers. Assess. 64, 21–50. 16367732 10.1207/s15327752jpa6401_2

[ref603] Cremers H. R. Demenescu L. R. Aleman A. Renken R. van Tol M. J. van der Wee N. J.. (2010). Neuroticism modulates amygdala—prefrontal connectivity in response to negative emotional facial expressions. Neuroimage 49, 963–970. 19683585 10.1016/j.neuroimage.2009.08.023

[ref12] Crossley S. A. Allen D. B. McNamara D. S. (2011). Text readability and intuitive simplification: a comparison of readability formulas. Reading Foreign Lang. 23, 84–101.

[ref13] Daly J. A. (2011). “Personality and interpersonal behavior,” in The SAGE Handbook of Interpersonal Communication. eds. Daly J. A. Knapp M. L. (Thousand Oaks, CA: Sage Publications, Inc).

[ref14] Dewaele J.-M. Furnham A. (1999). Extraversion: the unloved variable in applied linguistic research. Lang. Learn. 49, 509–544. doi: 10.1111/0023-8333.00098

[ref15] Dewaele J.-M. Furnham A. (2000). Personality and speech production: a pilot study of second language learners. Personal. Individ. Differ. 28, 355–365. doi: 10.1016/S0191-8869(99)00106-3

[ref606] Eysenck M. W. Derakshan N. Santos R. Calvo M. G. (2007). Anxiety and cognitive performance: attentional control theory. Emotion 7:336. 17516812 10.1037/1528-3542.7.2.336

[ref16] Funder D. C. (1995). On the accuracy of personality judgment: a realistic approach. Psychol. Rev. 102, 652–670. doi: 10.1037/0033-295X.102.4.652, PMID: 7480467

[ref17] Funder D. C. Colvin C. R. (1988). Friends and strangers: acquaintanceship, agreement, and the accuracy of personality judgment. J. Pers. Soc. Psychol. 55, 149–158. doi: 10.1037/0022-3514.55.1.149, PMID: 3418488

[ref18] Garcia S. Stinson L. Ickes W. Bissonnette V. Briggs S. R. (1991). Shyness and physical attractiveness in mixed-sex dyads. J. Pers. Soc. Psychol. 61, 35–49. doi: 10.1037/0022-3514.61.1.35

[ref602] Gifford R. Hine D. W. (1994). The role of verbal behavior in the encoding and decoding of interpersonal dispositions. J. Res. Pers. 28, 115–132.

[ref19] Gill A. J. (2003). Personality and language: The projection and perception of personality in computer-mediated communication (Doctoral dissertation, University of Edinburgh).

[ref705] Gill A. Oberlander J. (2003). “Looking forward to more extraversion with n-grams,” in Determination of Information and Tenor in Texts: Multiple Approaches to Discourse, 2003, 125–137.

[ref20] Gosling S. D. Rentfrow P. J. Swann W. B. Jr. (2003). A very brief measure of the big-five personality domains. J. Res. Pers. 37, 504–528. doi: 10.1016/S0092-6566(03)00046-1

[ref21] Gunning R. (1969). The fog index after twenty years. J. Bus. Commun. 6, 3–13. doi: 10.1177/002194366900600202

[ref22] Harrison J. S. Thurgood G. R. Boivie S. Pfarrer M. D. (2019). Measuring CEO personality: developing, validating, and testing a linguistic tool. Strateg. Manag. J. 40, 1316–1330. doi: 10.1002/smj.3023

[ref23] Hirsh J. B. Peterson J. B. (2009). Personality and language use in self-narratives. J. Res. Pers. 43, 524–527. doi: 10.1016/j.jrp.2009.01.006

[ref24] Höweler M. (1972). Diversity of word usage as a stress indicator in an interview situation. J. Psycholinguist. Res. 1, 243–248. doi: 10.1007/BF01074440 24197683

[ref25] Hutto C. J. Gilbert E. E. (2014). “VADER: a parsimonious rule-based model for sentiment analysis of social media text.” in *Proceedings of the international AAAI conference on web and social media.* 8, 216–225.

[ref26] Ireland M. E. Mehl M. R. (2014). “Natural language use as a marker of personality,” in The Oxford handbook of language and social psychology. ed. Holtgraves T. M. (Oxford: Oxford University Press), 201–237.

[ref609] Ireland M. E. Slatcher R. B. Eastwick P. W. Scissors L. E. Finkel E. J. Pennebaker J. W. (2011). Language style matching predicts relationship initiation and stability. Psychol. Sci. 22, 39–44. 21149854 10.1177/0956797610392928

[ref601] Katz L. Dalby J. T. (1981). Computer and manual administration of the Eysenck Personality Inventory. J. Clin. Psychol. 37, 586–588. 7263884 10.1002/1097-4679(198107)37:3<586::aid-jclp2270370324>3.0.co;2-8

[ref610] Kern M. L. Eichstaedt J. C. Schwartz H. A. Dziurzynski L. Ungar L. H. Stillwell D. J.. (2014). The online social self: An open vocabulary approach to personality. Assessment 21, 158–169. 24322010 10.1177/1073191113514104

[ref27] Lee C. H. Kim K. Seo Y. S. Chung C. K. (2007). The relations between personality and language use. J. Gen. Psychol. 134, 405–413. doi: 10.3200/GENP.134.4.405-414 18183737

[ref28] Levine J. M. Moreland R. L. (2012). “A history of small group research,” in Handbook of the History of Social Psychology. eds. J. M. Levine and R. L. Moreland (Psychology Press), 382–404.

[ref29] Loughran T. McDonald B. (2011). When is a liability not a liability? Textual analysis, dictionaries, and 10-Ks. J. Financ. 66, 35–65. doi: 10.1111/j.1540-6261.2010.01625.x

[ref30] Mairesse F. Walker M. A. Mehl M. R. Moore R. K. (2007). Using linguistic cues for the automatic recognition of personality in conversation and text. J. Artif. Intell. Res. 30, 457–500. doi: 10.1613/jair.2349

[ref31] Mc Laughlin G. H. (1969). SMOG grading-a new readability formula. J. Read. 12, 639–646.

[ref32] McCrae R. R. Costa P. T. (2004). A contemplated revision of the NEO five-factor inventory. Personal. Individ. Differ. 36, 587–596. doi: 10.1016/S0191-8869(03)00118-1

[ref33] McCrae R. R. Costa P. T. Jr. (1983). Joint factors in self-reports and ratings: neuroticism, extraversion and openness to experience. Personal. Individ. Differ. 4, 245–255.

[ref34] McCrae R. R. John O. P. (1992). An introduction to the five-factor model and its applications. J. Pers. 60, 175–215. doi: 10.1111/j.1467-6494.1992.tb00970.x, PMID: 1635039

[ref35] Mehl M. R. Gosling S. D. Pennebaker J. W. (2006). Personality in its natural habitat: manifestations and implicit folk theories of personality in daily life. J. Pers. Soc. Psychol. 90, 862–877. doi: 10.1037/0022-3514.90.5.862, PMID: 16737378

[ref36] Mehl M. R. Pennebaker J. W. (2003). The sounds of social life: a psychometric analysis of students’ daily social environments and natural conversations. J. Pers. Soc. Psychol. 84, 857–870. doi: 10.1037/0022-3514.84.4.857, PMID: 12703653

[ref37] Moffitt K. C. Giboney J. S. Ehrardt E. Burgoon J. K. Nunamaker J. F. Jr. (2012). “Structured programming for linguistic cue extraction (SPLICE),” in Proceedings of the HICSS-45 Rapid Screening Technologies, Deception Detection and Credibility Assessment Symposium. eds. Jensen M. L. Meservy T. O. Burgoon J. K. Nunamaker J. F. Jr. (Maui, HI), 103–108.

[ref38] Montag C. Elhai J. D. (2019). A new agenda for personality psychology in the digital age? Personal. Individ. Differ. 147, 128–134. doi: 10.1016/j.paid.2019.03.045

[ref39] Moreno J. D. Martínez-Huertas J. Á. Olmos R. Jorge-Botana G. Botella J. (2021). Can personality traits be measured analyzing written language? A meta-analytic study on computational methods. Personal. Individ. Differ. 177:110818. doi: 10.1016/j.paid.2021.110818

[ref40] Muris P. Roelofs J. Rassin E. Franken I. Mayer B. (2005). Mediating effects of rumination and worry on the links between neuroticism, anxiety and depression. Personal. Individ. Differ. 39, 1105–1111. doi: 10.1016/j.paid.2005.04.005

[ref41] Niederhoffer K. G. Pennebaker J. W. (2002). Linguistic style matching in social interaction. J. Lang. Soc. Psychol. 21, 337–360. doi: 10.1177/026192702237953

[ref607] Nowson S. (2006). The Language of Weblogs: A study of genre and individual differences.

[ref608] Park H. S. Guan X. (2006). The effects of national culture and face concerns on intention to apologize: A comparison of the USA and China. J. Intercult. Commun. Res. 35, 183–204. doi: 10.1080/17475750601026933

[ref42] Pennebaker J. W. Boyd R. L. Jordan K. Blackburn K. (2015). The development and psychometric properties of LIWC-2015. Austin, TX: University of Texas at Austin.

[ref43] Pennebaker J. W. King L. A. (1999). Linguistic styles: language use as an individual difference. J. Pers. Soc. Psychol. 77, 1296–1312. doi: 10.1037/0022-3514.77.6.1296, PMID: 10626371

[ref44] Pentland S. J. Spitzley L. A. Chen X. Burgoon J. K. Nunamaker J. F. Jr. (2021). “Behavioral indicators of dominance in an adversarial group negotiation game,” in Detecting Trust and Deception in Group Interaction. Terrorism, Security, and Computation. eds. Subrahmanian V. S. Dunbar N. E. Burgoon J. K. (Springer), 99–122.

[ref45] Roccas S. Sagiv L. Schwartz S. H. Knafo A. (2002). The big five personality factors and personal values. Personal. Soc. Psychol. Bull. 28, 789–801. doi: 10.1177/0146167202289008

[ref46] Schnurr P. P. Rosenberg S. D. Oxman T. E. Tucker G. J. (1986). A methodological note on content analysis: estimates of reliability. J. Pers. Assess. 50, 601–609. doi: 10.1207/s15327752jpa5004_7, PMID: 16367429

[ref47] Singelis T. M. Triandis H. C. Bhawuk D. P. Gelfand M. J. (1995). Horizontal and vertical dimensions of individualism and collectivism: A theoretical and measurement refinement. Cross-Cult. Res. 29, 240–275. doi: 10.1177/106939719502900302

[ref49] Van Scotter J. R. Motowidlo S. J. (1996). Interpersonal facilitation and job dedication as separate facets of contextual performance. J. Appl. Psychol. 81, 525–531. doi: 10.1037/0021-9010.81.5.525

[ref50] Van Swol L. M. Kane A. A. (2019). Language and group processes: an integrative, interdisciplinary review. Small Group Res. 50, 3–38. doi: 10.1177/1046496418785019

[ref51] Wiener M. Mehrabian A. (1968). Language Within Language: Immediacy, a Channel in Verbal Communication. New York: Ardent Media.

[ref52] Witt L. A. Ferris G. R. (2003). Social skill as moderator of the conscientiousness-performance relationship: convergent results across four studies. J. Appl. Psychol. 88, 809–820. doi: 10.1037/0021-9010.88.5.809, PMID: 14516246

[ref53] Yarkoni T. (2011). Personality in 100,000 words: a large-scale analysis of personality and word use among bloggers. J. Res. Pers. 44, 363–373. doi: 10.1016/j.jrp.2010.04.001, PMID: 20563301 PMC2885844

[ref54] Zhou L. Sung Y. Zhang D. (2013). Deception performance in online group negotiation and decision making: the effects of deception experience and deception skill. Group Decis. Negot. 22, 153–172. doi: 10.1007/s10726-012-9303-9

