# Supplementary figures and images for: Linguistic measures of personality in group discussions
Source: Front Psychol. 2022 Sep 16;13:887616. doi: 10.3389/fpsyg.2022.887616 (PMC9523152; doi:10.3389/fpsyg.2022.887616)

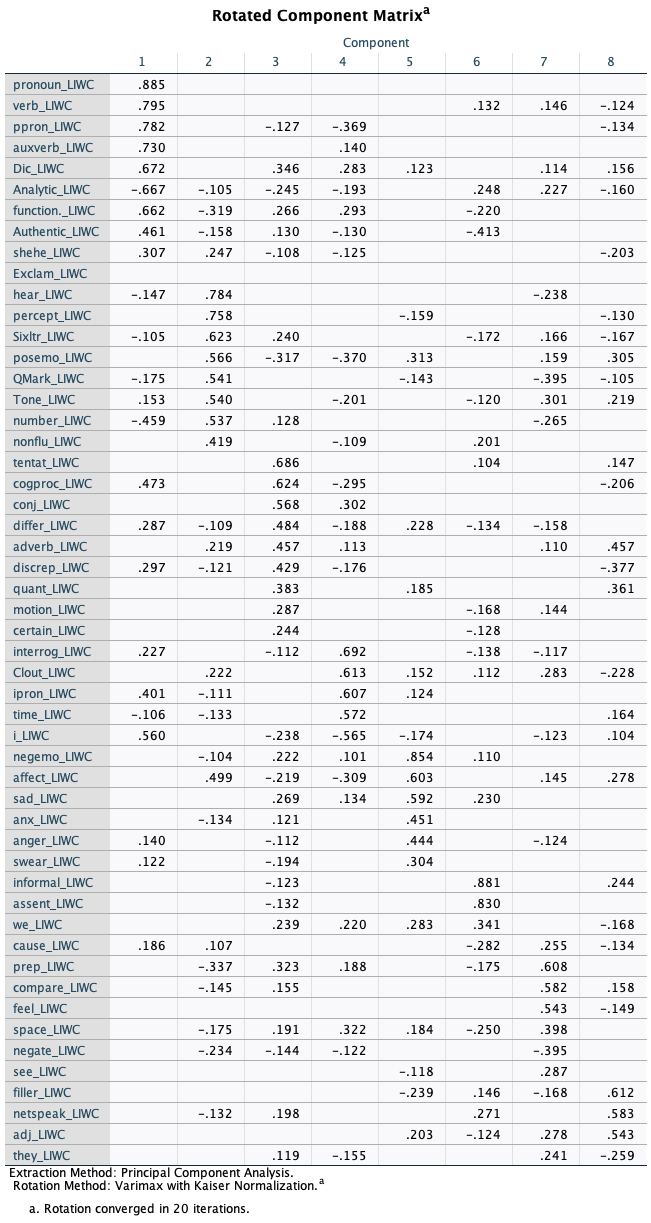

Supplement: Supplementary file 2 [file Table_2.DOCX]
