# Supplementary material for: Linguistic measures of personality in group discussions
Source: Front Psychol. 2022 Sep 16;13:887616. doi: 10.3389/fpsyg.2022.887616 (PMC9523152; doi:10.3389/fpsyg.2022.887616)
Supplement: Supplementary file 3 [file Table_4.docx]

To address the concern on the high number of features, adjustments for multiple comparisons are performed to re-evaluate the regression models. Among various methods, we select the Bonferroni correction (Bland & Altman, 1995) and the Benjamini-Hochberg (BH) adjustment (Benjamini & Hochberg, 1995). Both methods are commonly used but the Bonferroni correction is much more conservative than the BH adjustment. By employing both methods, we intend to demonstrate how many of the original findings still hold when different statistical criteria are applied. Table 1 compares the number of significant linguistic features for each personality trait when different adjustments are implemented. No null hypothesis was rejected when the Bonferroni correction was applied. That is, all originally significant relationships became invalid. However, the Bonferroni correction is notable for its conservative nature and has been found to increase Type II error. When the significance cutoff was relaxed to 0.1, the relationship between a few linguistic features and the personality dimensions became significant. Furthermore, when the more powerful BH adjustment was in use, more null hypotheses were rejected, and more significant relationships retained. This pattern was more pronounced for the LIWC features than for the SPLICE features.

In summary, we considered the risk of repeated analysis. However, even after conducting p-value adjustments, various verbal features were still identified as significant personality indicators, which shows robustness in our findings. Future analysis may focus only on the content or style aspects of language to reduce the feature space and mitigate the multiple comparison issue.

| LIWC Regression Models | | | | | | |
| --- | --- | --- | --- | --- | --- | --- |
| Methods | Extraversion | Agreeableness | Conscientiousness | Neuroticism | Openness | Sum |
| None | 14 | 5 | 7 | 6 | 3 | 35 |
| BF (p<0.05) | 0 | 0 | 0 | 0 | 0 | 0 |
| BF (p<0.1) | 2 | 1 | 0 | 0 | 0 | 3 |
| BH (p<0.05) | 10 | 2 | 2 | 6 | 3 | 23 |
| SPLICE Regression Models | | | | | | |
| None | 5 | 3 | 4 | 4 | 1 | 17 |
| BF (p<0.05) | 0 | 0 | 0 | 0 | 0 | 0 |
| BF (p<0.1) | 0 | 1 | 0 | 0 | 0 | 1 |
| BH (p<0.05) | 2 | 0 | 1 | 1 | 0 | 4 |

Notes: BF, Bonferroni correction; BH, Benjamini-Hochberg (BH) adjustment

**Table 1. Number of significant linguistic features under different p-value adjustments**

**References**

Bland, J. M., & Altman, D. G. (1995). Multiple significance tests: the Bonferroni method. *Bmj*, *310*(6973), 170.

Benjamini, Y., & Hochberg, Y. (1995). Controlling the false discovery rate: a practical and powerful approach to multiple testing. *Journal of the Royal statistical society: series B (Methodological)*, *57*(1), 289-300.
